# Enhanced anti-breast cancer efficacy of co-delivery liposomes of docetaxel and curcumin

**DOI:** 10.3389/fphar.2022.969611

**Published:** 2022-10-17

**Authors:** Xi Ye, Xin Chen, Ruixi He, Wangyang Meng, Weidong Chen, Fengling Wang, Xiangyun Meng

**Affiliations:** ^1^ Department of Pharmacy, The Second People’s Hospital of Hefei, Hefei, China; ^2^ Hefei Hospital Affiliated to Anhui Medical University, Hefei, China; ^3^ Hefei Hospital Affiliated to Bengbu Medical College, Hefei, China; ^4^ Department of Pharmacy, Anhui Provincial Crops Hospital, Hefei, China; ^5^ Anhui University of Chinese Medicine, Hefei, China; ^6^ Institute of Drug Metabolism, School of Pharmaceutical Sciences, Anhui University of Chinese Medicine, Hefei, China; ^7^ Union Hospital, Tongji Medical College, Huazhong University of Science and Technology, Wuhan, China

**Keywords:** co-delivery, curcumin, docetaxel, breast cancer, liposomes

## Abstract

The successful treatment of breast cancer is hampered by toxicity to normal cells, impaired drug accumulation at the tumor site, and multidrug resistance. We designed a novel multifunctional liposome, CUR-DTX-L, to co-deliver curcumin (CUR) and the chemotherapeutic drug docetaxel (DTX) for the treatment of breast cancer in order to address multidrug resistance (MDR) and the low efficacy of chemotherapy. The mean particle size, polydispersity index, zeta potential, and encapsulation efficiency of CUR-DTX-L were 208.53 ± 6.82 nm, 0.055 ± 0.001, −23.1 ± 2.1 mV, and 98.32 ± 2.37%, respectively. An *in vitro* release study and CCK-8 assays showed that CUR-DTX-L has better sustained release effects and antitumor efficacy than free drugs, the antitumor efficacy was verified by MCF-7 tumor-bearing mice, the CUR-DTX-L showed better antitumor efficacy than other groups, and the *in vivo* pharmacokinetic study indicated that the plasma concentration–time curve, mean residence time, and biological half-life time of CUR-DTX-L were significantly increased compared with free drugs, suggesting that it is a promising drug delivery system for the synergistic treatment of breast cancer.

## 1 Introduction

Cancer remains the second leading cause of death worldwide, with breast cancer being the most commonly diagnosed malignancy and the leading cause of cancer-related deaths in women worldwide, despite the fact that extensive research is being conducted worldwide to combat this dreadful and lethal disease (GBD 2017 Causes of Death Collaborators, 2018; Schmidt et al., 2017). In addition, the incidence of breast cancer, which affects one in eight women, is rising. Traditional breast cancer treatments include surgery, radiation, and chemotherapy, with chemotherapy being the most prevalent. Docetaxel (DTX), an artificial semi-synthetic yew chemical generated from the needles of European yew, is one of the first-line medications for breast cancer treatment that inhibits the proliferation of cancer cells by interfering with their synthesis, migration, and division (Shi et al., 2015; Hua et al., 2017). However, the side effects caused by docetaxel have considerably overshadowed its clinical use. First, like most other classical chemotherapeutic drugs, DTX is distributed throughout the body in a non-specific manner, and second, due to the poor solubility of DTX in water, it can lead to adverse drug reactions (Persohn et al., 2005). In addition, multidrug resistance (MDR) has already been a major problem in clinical treatment that limits the efficacy of DTX; MDR can lead to rapid drug elimination and chemotherapy failure, and it may be associated with multiple mechanisms (Bukowski et al., 2020), among which drug efflux transporters are the main cause of MDR (Gote et al., 2021), which remove DTX from tumor cells and reduce the accumulation of drug-resistant cells. Therefore, in order to improve the antitumor efficacy and reduce side effects caused by the nonspecific delivery of DTX, it is imperative that the preparation and development of novel techniques be expedited (Kaushik et al., 2020).

In recent years, combination therapy of simultaneous administration of numerous medications has proven to be superior to the use of a single drug in clinical settings, thereby reducing the occurrence of MDR (Berríos-Caro et al., 2021). For instance, it is an effective strategy to alter the efficacy of anticancer agents by co-administration of reversal agents. Curcumin (CUR), a bioactive ingredient extracted from the rhizome of the herb turmeric, which has been recognized as a safe food additive by the FDA, is also being widely used in the prevention and treatment of a variety of cancers, including preclinical studies in breast cancer, colorectal cancer, stomach cancer, liver cancer, esophageal cancer, lung cancer, brain cancer, and leukemia (Chen et al., 2016; Hesari et al., 2019; Hong et al., 2019; Wong et al., 2019; Guo P. et al., 2020; Angeline et al., 2020; Schmidt et al., 2020). Furthermore, CUR is also well known to downregulate P-gp, MRP-1, and other components that contribute to MDR (Abouzeid et al., 2014). It has been demonstrated that the growth inhibitory and cell death-inducing effects of CUR and its analogs are not reduced by drug resistance in breast cancer MCF-7 cells (Labbozzetta et al., 2009). Growing interest has been focused on CUR-mediated combination of drug delivery systems. Studies have shown that the combination of CUR and chemotherapy drugs like paclitaxel and doxorubicin may improve treatment efficacy (Wang et al., 2019; Zhao et al., 2019). However, despite the promise of its pharmacological capabilities, the therapeutic application of CUR is now constrained by its insolubility in water and low bioavailability. If CUR is simply combined with other anti-cancer drugs to work directly on cancer cells, it will not achieve its full effect.

Nanoparticle drug delivery methods, such as liposomes, micelles, and nanoproducts, have gained increasing interest over the past decades due to their numerous advantages (Wang et al., 2011; Ran et al., 2014; Li et al., 2020). Among these, liposomes are considered to be a powerful drug delivery system due to their structural versatility, biocompatibility, biodegradability, non-toxicity, and non-immunogenicity (Mathiyazhakan et al., 2018). Liposomes are phospholipid vesicles formed by one or more concentric lipid bilayers surrounding discrete aqueous cavities. The liposome system has the unique ability to trap lipophilic and hydrophilic compounds, allowing a variety of drugs to be encapsulated by these vesicles (Sercombe et al., 2015). The process of medication release from liposomes is the disintegration of the phospholipid bilayer, and pharmaceuticals encapsulated in liposomes can be simultaneously released to exhibit their effectiveness (Yu et al., 2009).

**FIGURE 1:**
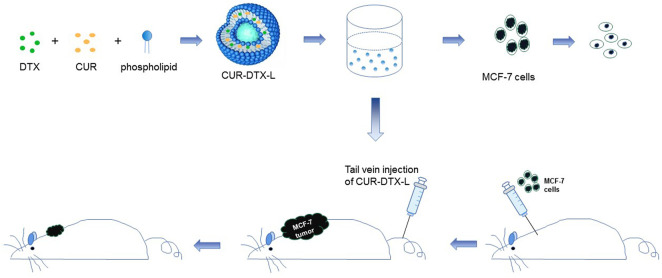
Schematic diagram of the assembly of CUR-DTX-L and its *in vitro* and *in vivo* efficacy tests. CUR-DTX-L was prepared by ethanol injection. The *in vitro* efficacy of CUR-DTX-L was investigated in the MCF-7 cell line, and its *in vivo* efficacy was investigated with an MCF-7 nude mouse model.

In this report, we designed a CUR and DTX co-delivery strategy with liposomes as nanocarriers to synergistically induce apoptosis in human breast cancer. We formulated CUR-DTX-L by the ethanol injection method, which enabled the co-delivery of DTX and CUR in a single system. CUR-DTX-L was characterized in terms of particle size and zeta potential, and the *in vivo* pharmacokinetics of this liposome was investigated. Moreover, *in vitro* and *in vivo* pharmacodynamic studies of CUR-DTX-L were performed to evaluate its efficacy for antitumor activity in MCF-7 cells. This system is expected to achieve the stable and controlled release and active targeting of DTX and CUR to liposomes, improve synergistic anticancer effects, and reduce toxicity (Figure 1).

## 2 Materials and methods

### 2.1 Materials

Docetaxel (DTX), curcumin (CUR), and cholesterol (Chol) of analytical grade were acquired from Sinopharm Chemical Reagent Co., Ltd. (Shanghai, China). The supplier of egg phospholipid was A.V.T. (Shanghai) Pharmaceutical Co., Ltd. Merck was contacted to acquire methanol and acetonitrile of HPLC quality (Darmstadt, Germany). The remaining reagents were all of analytical grade.

### 2.2 Cells and animals

MCF-7 cells (Cell Bank of Shanghai Institute of Cell Biology, China) were cultured in DMEM supplemented with 10% (v/v) fetal bovine serum (FBS, Gibco), 4 ml glutamine, 4,500 mg/L glucose, 100 units/ml penicillin, and 100 units/ml streptomycin at 37°C in a humidified 5% CO_2_ environment (Zhang et al., 2017).

Anhui Medical University’s Experimental Animal Center supplied 220 ± 20 g of 5-week-old Sprague–Dawley (SD) rats and BALB/c nude female mice that were in good health (Hefei, China). All animals were housed in vented cages at a controlled temperature of 24°C–26°C and relative humidity of 55%–65%, and they were permitted to consume regular feed and drink water *ad libitum* (Wu et al., 2016). All protocols involving experimental animals were performed in accordance with the guidelines evaluated and approved by the Institutional Animal Ethics Committee of Anhui Medical University (Hefei, China).

### 2.3 Preparation of liposomes

The CUR-DTX liposomes (CUR-DTX-L) were prepared by the ethanol injection method (Du and Deng, 2006; Vitor et al., 2017). Using single-factor experiments, the formulation process was optimized, and the optimization procedure is given as follows. Briefly, CUR (2 mg), DTX (4 mg), cholesterol (30 mg), and soybean phospholipid (120 mg) were dissolved in ethanol (3 ml) to generate the organic phase, and the mixed organic solution was deposited in a 55°C water bath. The solution was then rapidly injected into the agitated aqueous phase (20 ml of double-distilled water, 800 rpm). The aqueous phase was then agitated for 3 h at 55°C using an electric magnetic stirrer (DF-1 Electric Stirrer, JinTan, China) to produce a homogenous emulsion.

Single CUR-loaded liposomes (CUR-L) were prepared using the same procedure as CUR-DTX-L but without the addition of DTX to the organic phase. The amount of soybean phospholipid and cholesterol was changed to 130 mg and 20 mg, respectively.

Single DTX-loaded liposomes (DTX-L) were prepared using the same method as CUR-DTX-L but without the addition of CUR to the organic phase. The amount of soybean phospholipid and cholesterol was changed to 130 mg and 20 mg, respectively.

### 2.4 Encapsulation efficiency

The encapsulation efficiency (EE) was determined by measuring the unencapsulated drug concentration and total drug concentration by the high-performance liquid chromatography (HPLC) method (Wang et al., 2020). The unencapsulated DTX and CUR were separated from liposomes by size exclusion chromatography using a Sephadex G-50 column (1.5 cm × 20.0 cm) with distilled water as the eluent. Briefly, the isolated CUR-DTX-L, DTX-L, and CUR-L were dissolved in methanol and determined by HPLC. The EE% could be calculated as follows:

EE(%)=WE/WT×100%,

where WE refers to the weight of CUR/DTX encapsulated in liposomes and WT refers to the weight of the total CUR/DTX in the formulation.

### 2.5 Characterization of liposomes

Mean particle size, zeta potential, and polydispersity index (PDI) of the liposomes were measured by dynamic light scattering (DLS) using a Zetasizer Nano ZS90 Malvern particle size analyzer (Malvern, United Kingdom) after dilution with distilled water. The instrument was calibrated using standard latex liposomes. All particle size measurements were carried out after the liposomes were diluted 20-fold in distilled water. The zeta potential measurements of the prepared liposomes can be performed without dilution. The experimental data represented the mean of three distinct outcomes (Wu et al., 2020).

### 2.6 *In vitro* release of liposomes

In combination with the chemical properties of DTX and CUR, PBS (pH = 7.4) was selected as the release medium, and the solubility of DTX and CUR was enhanced by the addition of 0.5% Tween 80 to achieve the condition of tank leakage in order to observe the release characteristics of liposomes *in vitro*. In this experiment, samples containing 0.5 mg of drugs were placed in dialysis membrane bags (21 mm, MVCO 8000-14400 Da), tied, and submerged in a beaker containing 100 ml of the release medium at (37 ± 1)°C. Separate samples were taken at regular intervals, and an equal volume of the release medium was added to ensure sedimentation conditions. After passing the samples through a 0.22-μm filter membrane, the amount of DTX and CUR was determined using the HPLC technique (Ji et al., 2006).

### 2.7 Pharmacokinetic studies

In this investigation, male and female rats were randomly separated into six equal groups, and formulations were injected intravenously through the tail vein (*n* = 6 per group). Before administration, DTX-L, CUR-L, and CUR-DTX-L were freshly prepared. Each group was injected with free CUR, free DTX, free DTX-CUR, CUR-L, DTX-L, and CUR-DTX-L, and the CUR and DTX doses were 1.0 mg/kg and 2.0 mg/kg, respectively. Approximately, 0.3 ml of blood was taken in heparinized microcentrifuge tubes *via* the retro-orbital under isoflurane anesthesia immediately following a pre-determined post-injection time point (3, 10, 30, 60, 120, 240, 360, 480, 600, and 720 min). After the blood samples were centrifuged at 3,000 rpm for 10 min, plasma (150 L) was extracted and kept at −20°C until further HPLC analysis (Sun et al., 2018). The pharmacokinetic parameters of DTX and CUR were estimated using the software program DAS 2.0 (Shanghai Bojin Medical Technology Co., Ltd., Shanghai, China).

### 2.8 Cell viability assays

To examine cell viability, the Cell Counting Kit-8 (CCK-8) approach was chosen (Zhang et al., 2020). MCF-7 breast cancer cells were cultured at a density of 5,000 cells per well in 96-well plates and cultured at 37°C in a humidified room with 5% CO_2_. After 24 h of culture, DTX-CUR-L, DTX-L, CUR-L, free DTX, and free CUR were applied to the adherent cells. Due to its limited water solubility, DMSO dissolves free DTX and CUR rapidly (final concentration of DMSO in medium ≤ 0.1%). The concentration gradients of DTX given to adherent cells were 25, 50, 75, 100, 120, 150, and 200 g/ml. The respective concentration gradients of CUR were 12.5, 25, 37.5, 50, 60, 75, and 100 g/ml. After 24 h of incubation at 37°C and 5% CO_2_, the culture media were withdrawn from each well, and the cells were washed twice with PBS. Each well received 10 μl of CCK-8 solution (it should be noted not to develop air bubbles during the addition process), and the culture plate was incubated for an additional hour. At 490 nm, absorbance values were measured using an enzyme-linked immunosorbent assay reader (BioTek elx800 microplate reader, United States). Cell viability was calculated as the percentage of absorbance in the wells of treated cells relative to that of untreated cells. Assuming 100% survival of untreated cells, the percentage of viable cells can be calculated (Tian et al., 2018). In this study, all treatments were evaluated in triplicate, and the results are expressed as the mean standard deviation. The sample’s half-maximal inhibitory concentration (IC_50_) value was calculated.

### 2.9 *In vivo* antitumor study

#### 2.9.1 Modeling and administration

MCF-7 cells were grown in DMEM media supplemented with 10% fetal bovine serum (FBS). When the cells reached the exponential growth phase, they are digested with 0.25% trypsin and spun at 1,200 rpm for 5 min. The serum from the cell precipitation was removed by rinsing it twice with PBS and resuspending the cells in PBS. Cells were counted under a microscope and diluted to 1×10^7^ cells/ml. Afterward, 10^6^ MCF-7 cells were implanted into the mammary fat pad close to the left axilla (Wang et al., 2015). After 10 days, the tumor-bearing mice were randomly divided into seven groups of three mice each: the saline group, CUR group, DTX group, CUR/DTX group, CUR-L group, DTX-L group, and CUR-DTX-L group. The groups received tail vein injections of corresponding medicines every third day four times, and the CUR and DTX doses were 0.1 mg/kg and 0.2 mg/kg, respectively. During therapy, each mouse was weighed, and the highest (D) and minimum 4) tumor diameters were measured with a caliper every other day. After the final injection, the mice were watched for a total of two treatment cycles. On day 14, all animals were sacrificed by cervical dislocation. Tumors and organs were removed, weighed, photographed, and then preserved in paraformaldehyde at 4% for subsequent studies.

#### 2.9.2 The analysis of tumor growth inhibition and weight

Using a caliper, the maximum (D) and minimum (d) diameters of the tumors were determined. The volume (V) of the tumor was computed using the formula: V = Dd2/2 (Gao et al., 2013). The tumor growth inhibition curves for the different groups are depicted using time as the horizontal axis and tumor volume as the vertical axis. Photographs of excised mouse tumors can directly indicate the efficacy of the drug. The weight of the tumor is a statistical measure of anticancer activity. Taking the tumor weight as the ordinate and the number of experimental groups as the abscissa, the differences in tumor weight between the groups were examined. According to the equation,

$$Tumor\;inhibition\;rate\;\left(IRT\%\right)=\frac{\left|\left(W\;control-W\;experiment\right)\right|}{W\;control}\times 100\%.$$

the tumor inhibition rate of each prescription drug was calculated, and its antitumor efficacy was evaluated.

#### 2.9.3 Histological examination and analysis

After washing the removed tumor with normal saline, it was fixed with 4% paraformaldehyde for more than 24 h, embedded in paraffin, stained with hematoxylin and eosin (H and E), and observed and examined at ×200 magnification using a microscope (He et al., 2010). The apoptosis of tumor cells was assessed by terminal deoxynucleotidyl transferase dUTP nick end labeling (TUNEL) stain labeling. The counterstain utilized was diaminobenzidine (DAB). In the TUNEL experiment, the apoptotic cell nuclei were stained brown. Tissue samples were routinely fixed with 4% paraformaldehyde, embedded in paraffin, stained with TUNEL, and sectioned (Nassir et al., 2019). Under a microscope, the sections were examined and photographed. The positive expression rates of apoptotic cells in the images of each group were compared.

### 2.10 Assessment of toxicity in MCF-7 tumor-bearing nude mice

Safety is mostly determined by observing the weight change of mice with tumors and the toxicity of drug-loaded micelles on the organs of mice by HE staining. The weight change curve is obtained by weighing and recording the weight of shaved mice every 2 days, and then, the time–weight curve is plotted. After removing the mouse’s heart, liver, spleen, lung, and kidney, the removed organs were washed with normal saline, fixed with 4% paraformaldehyde, embedded in paraffin, stained with HE, and examined and studied at ×200 magnification using a microscope.

### 2.11 Statistical analysis

All data are provided as the mean ± SD. *T*-tests were used to compare two groups, while one-way analyses of variance (ANOVA) were utilized to compare multiple groups. *p*-values of 0.05 were statistically significant. Using SPSS 18.0 software, statistical analysis was performed.

## 3 Results

### 3.1 Characterization of liposomes

The properties of the drugs encapsulated in liposomes were analyzed. In this study, a nanoscale drug delivery system was used to encapsulate DTX and CUR, both of which are poorly water-soluble anticancer drugs (Frank et al., 2019). Drugs with strong lipid solubility and water solubility are typically loaded passively into liposomes. The injection method and thin-film ultrasonic dispersion method are the primary preparation methods. According to preliminary experiments, although the thin-film dispersion method could complete the preparation of liposomes in a short amount of time, the system was cloudy, the particle size distribution was uneven, the stability was poor, and the amount of residual organic solvents exceeded the standard. Injection-prepared liposomes with a high EE percentage, tiny particle size, uniform dispersion, and good stability met the parameters for liposome preparation. According to Table 1, the EE values of CUR-DTX-L, DTX-L, and CUR-L synthesized using this approach were 98.32 ± 2.37%, 95.63 ± 3.65%, and 92.78 ± 3.07%, respectively.

**TABLE 1:** Characterization of different formulations (*n* = 3).

Parameter	Formulation		
	CUR-DTX-L	DTX-L	CUR-L
Size (nm)	208.53 ± 6.82	239.32 ± 4.95	212.95 ± 4.57
PDI	0.055 ± 0.001	0.134 ± 0.005	0.200 ± 0.012
Zeta potential (mV)	−23.1 ± 2.1	−20.5 ± 1.8	−19.4 ± 1.8
EE (%)	98.32 ± 2.37	95.63 ± 3.65	92.78 ± 3.07

The mean particle size and PDI of liposomes have a substantial influence on the EE percentage, stability, half-life *in vivo*, and targeted selectivity. Consequently, determining the average particle size is crucial for assessing the quality of liposomes. PDI is an index for determining particle size distribution uniformity (Guo D. et al., 2020). The lower the PDI number, the more homogeneous the particle size distribution will be. The average particle size of DTX-CUR-L, DTX-L, and CUR-L, as shown in Table 1, is 208.53 ± 6.82 nm, 239.32 ± 4.95 nm, and 212.95 ± 4.57 nm, respectively. The combination of DTX-L and CUR-L did not significantly alter the dimensions of either material. Moreover, the formulations showed PDI values of approximately 0.055 ± 0.001, 0.134 ± 0.005, and 0.200 ± 0.012, indicating that the particle size distribution in these three formulations is narrow and the particle size is relatively uniform.

The zeta potential formed by the double electric layer on the liposome particle’s surface is an additional essential component influencing its stability and action *in vivo*. It is generally believed that the bigger the absolute value of the zeta potential, the greater will be the electrostatic repulsion force between liposome particles, which can prevent the agglomeration and sedimentation of each particle in the dispersion system, and the physical stability is good (Alvebratt et al., 2020). Table 1 shows that all liposomes are negatively charged (−23.1 ± 2.1 mV for CUR-DTX-L, −20.5 ± 1.8 mV for DTX-L, and −19.4 ± 1.8 mV for CUR-L), indicating that the repulsive force prevents particles from aggregating, resulting in greater stability.

### 3.2 *In vitro* release kinetics

Due to the preliminary laboratory foundation, this experiment followed the preliminary experiment in the selection of a preparation method and the research of fundamental prescription, and on this basis, liposomes with a relatively high encapsulation rate and stability were obtained (Yu et al., 2017). The rate of drug release from the liposome is proportional to its permeability. *In vitro* release study circumstances can be utilized to imitate the physiological environment in the body, and the permeability and release rate of the medication can be initially understood, providing a firm foundation for future pharmacokinetics research. In PBS (pH = 7.4) without an additional surfactant, the solubility of DTX and CUR is exceedingly low, and it is challenging to achieve sink conditions. In this experiment, therefore, PBS containing 0.5% Tween 80 was selected as the release medium.

**FIGURE 2:**
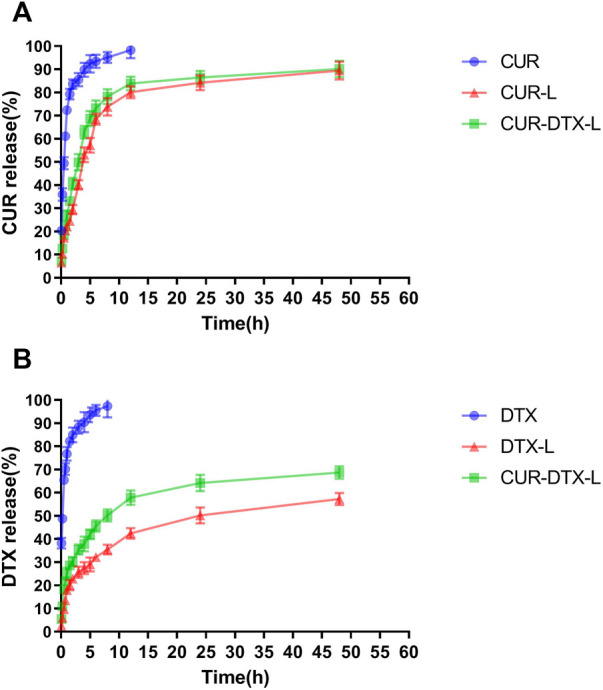
**(A)** Release profiles of DTX from DTX, DTX-L, and CUR-DTX-L in PBS (pH 7.4) at 37°C (*n* = 6). **(B)** Release profiles of CUR from CUR, CUR-L, and CUR-DTX-L in PBS (pH 7.4) at 37°C (*n* = 6).

We studied the rate of DTX and CUR release from liposomes and free drug solutions for up to 48 h in order to determine the release profile of the formulations and free medication. Figure 2 shows that liposomes have a slow-release feature. The results revealed that, compared to the free DTX and CUR solution, liposome preparations can delay the release of DTX and CUR *in vitro* and retain higher concentrations for a longer period of time. As shown in Figure 2A, nearly 100% of the free DTX was measured within the first 24 h; however, only 59.27% DTX and 68.74% DTX were released from DTX-L and CUR-DTX-L, respectively. Figure 2B shows that almost all of the free CUR are measured within the first 12 h, whereas CUR in the formulation is not completely released until 48 h. Moreover, compared with the rapid release of the free drug group, *in vitro* release studies have shown that liposome formulations exhibit slow drug release characteristics without initial bursts, which may be due to the fact that affinity of the drug held by small fragments of the liposome membrane lipid properties, as well as drugs encapsulated in lipid membranes, is mainly released by dissolution and diffusion of the lipid bilayer (Cheng et al., 2019). In addition, the encapsulation of liposomes changed the *in vitro* release rate of DTX and CUR, suggesting the possibility of the sustained release of the preparation *in vivo*, and this sustained-release mode may be beneficial in that the drug is not released during normal vascular circulation, and most of the embedded drugs can be delivered directly to the tumor site.

### 3.3 Pharmacokinetic studies

**FIGURE 3:**
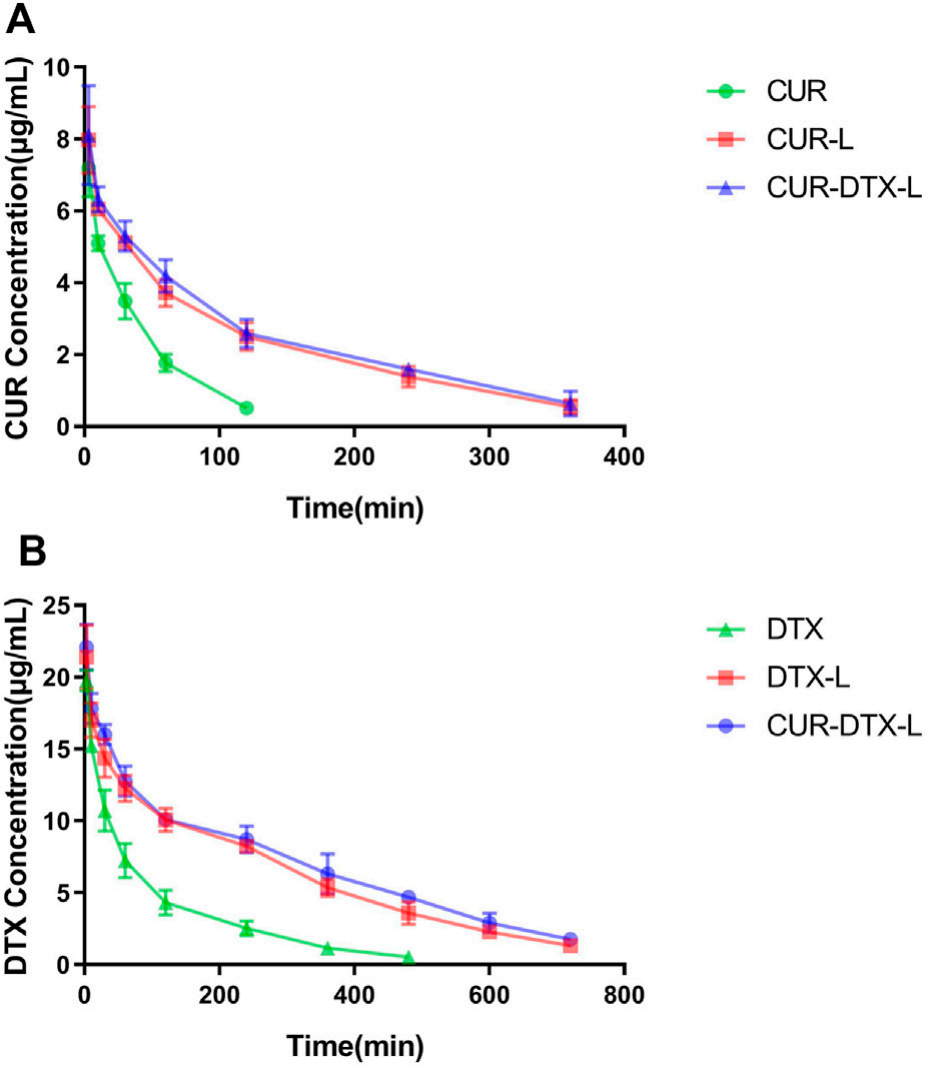
**(A)** Plasma concentrations vs. time curves of DTX in SD rats after intravenous injection of DTX, DTX-L, and CUR-DTX-L (*n* = 6). **(B)** Plasma concentrations vs. time curves of CUR in SD rats after intravenous injection of CUR, CUR-L, and CUR-DTX-L (*n* = 6).

Pharmacokinetics is mainly a quantitative study of the process (absorption, distribution, metabolism, and excretion) of drugs in the body and uses mathematical principles and methods to elaborate the dynamic laws of drugs in the body (Cheng et al., 2019). In this work, rats were given intraperitoneal dosages of 2.0 mg/kg (DTX) and 1.0 mg/kg (CUR). *In vivo* pharmacokinetic investigations were performed on CUR-L, DTX-L, and CUR-DTX-L, with free DTX and free CUR solutions serving as controls. The principal pharmacokinetic characteristics of the experiment are given in Tables 2, 3, and the plasma drug concentration–time curves are shown in Figure 3. As shown in Table 2, it can be clearly observed that compared to free DTX, the DTX-L and CUR-DTX-L formulations exhibit significantly higher T_1/2_ (106.6 ± 12.2, 188.1 ± 25.1, and 214.8 ± 24 min, respectively), AUC (1750.7 ± 159.3, 4565.1 ± 335.1, and 5050.9 ± 401 min·μg/ml, respectively), and MRT (147.2 ± 5.0, 287.4 ± 19.5, and 322.5 ± 17.0 min, respectively), which indicated that the DTX-L and CUR-DTX-L formulations can significantly improve the relative bioavailability of DTX. Similar results were observed with CUR (Table 3), where the main pharmacokinetic parameters were significantly increased in formulation groups compared to free CUR. In addition, the pharmacokinetic characteristics and concentration–time curves of DTX and CUR indicate that CUR-DTX-L had superior pharmacokinetic performance in rats than DTX-L or CUR-L. This may be due to the interaction between CUR and DTX in rats, resulting in a delayed metabolism of both compounds *in vivo* (Rao et al., 2020). In addition, the longer circulation period of liposomes could limit the absorption of proteins and the uptake of RES, hence preventing the nanoparticles from being rapidly eliminated (Sun et al., 2017).

**TABLE 2:** Pharmacokinetic parameters of CUR in formulations and free drugs (*n* = 6).

Parameter	Mean ± SD		
	CUR-DTX-L	CUR-L	Free CUR
T1/2 (min)	109.2 ± 19.7*	108.7 ± 22.5*	33.6 ± 3.3
Cmax (μg/ml)	8.1 ± 1.4*	8.0 ± 0.9*	7.2 ± 0.8
AUC(0-∞) (min·μg/mL)	901.2 ± 73.9*	834.6 ± 84.2*	287.2 ± 15.5
CL (ml/min/kg)	2.0 ± 0.3*	2.2 ± 0.3*	6.4 ± 0.3
MRT (min)	157.8 ± 32.9*	152.7 ± 26.8*	46.7 ± 3.3

*p < 0.05, compared with free CUR.

**TABLE 3:** Pharmacokinetic parameters of DTX in formulations and free drugs (*n* = 6).

Parameter	Mean ± SD		
	CUR-DTX-L	DTX-L	Free DTX
T_1/2_ (min)	214.8 ± 24*	188.1 ± 25.1*	106.6 ± 12.2
C_max_ (μg/ml)	22.1 ± 1.6*	21.4 ± 2.2*	19.8 ± 0.7
AUC_(0-∞)_ (min·μg/mL)	5050.9 ± 401*	4565.1 ± 335.1*	1750.7 ± 159.3
CL (ml/min/kg)	0.4 ± 0.0*	0.4 ± 0.0*	1.1 ± 0.1
MRT (min)	322.5 ± 17.0*	287.4 ± 19.5*	147.2 ± 5.0

*p < 0.05, compared with free DTX.

### 3.4 *In vitro* cytotoxicity studies

**FIGURE 4:**
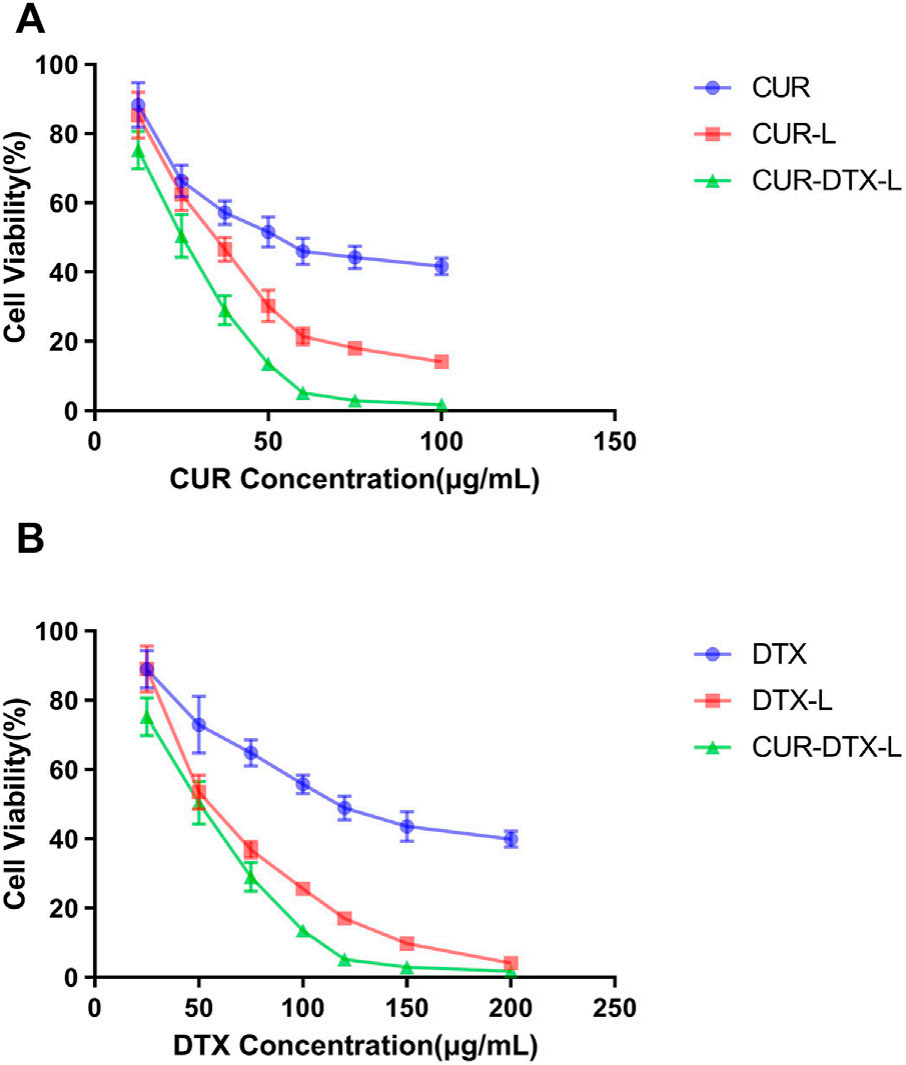
**(A)** MCF-7 cell viability vs. CUR concentration of CUR, CUR-L, and CUR-DTX-L (*n* = 3). **(B)** MCF-7 cell viability vs. DTX concentration of DTX, DTX-L, and CUR-DTX-L (*n* = 3).

CCK-8 assay was used to investigate the *in vitro* cytotoxicity of CUR-DTX-L, DTX-L, CUR-L, and free medicines on MCF-7 cells. Figure 4 shows the cell viability curves, from which it can be seen that free DTX and drug-loaded formulations inhibited cell proliferation dose-dependently in both cell types. In drug-loaded formulations, the vitality of MCF-7 cells was much lower than that of free DTX. After 24 h of incubation, seven distinct drug concentrations (DTX: 25, 50, 75, 100, 120, 150, and 200 g/ml; CUR: 12.5, 25, 37.5, 50, 60, 75, and 100 g/ml) exhibited significant differences in cell viability when compared to free drugs. In addition, the results demonstrated that CUR-DTX-L inhibited the proliferation of MCF-7 cells more effectively than DTX-L. The IC_50_ data (Table 4) demonstrated that CUR-DTX-L has the highest cytotoxicity compared to the other groups. On the one hand, liposomes can enhance the toxicity of medications, and on the other hand, CUR can reverse cell resistance and enhance the cytotoxicity of the treatment (Rejinold et al., 2018). Therefore, CUR-DTX-L may provide a novel insight into breast cancer treatment and palliation.

**TABLE 4:** IC_50_ values of formulations and free drugs in MCF-7 cells measured by the CCK-8 assay (*n* = 3).

IC_50_ (μg/ml)	Formulation				
	CUR-DTX-L	DTX-L	CUR-L	Free DTX	Free CUR
DTX	45.71 ± 3.15	57.81 ± 4.06	NA	125.03 ± 5.27	NA
CUR	22.86 ± 1.58	NA	36.24 ± 3.16	NA	53.26 ± 4.22

NA, not applicable.

The IC_50_ values of various formulations are summarized in Table 4. It is obvious that CUR-DTX-L showed lower IC_50_ values (for DTX: 45.71 ± 3.15 μg/ml and for CUR: 22.86 ± 1.58 μg/ml) than DTX-L (57.81 ± 4.06 μg/ml), CUR-L (36.24 ± 3.16 μg/ml), and free drugs (for DTX: 125.03 ± 5.27 μg/ml and for CUR: 53.26 ± 4.22 μg/ml). These results indicate that CUR enhances DTX-mediated apoptosis in MCF-7 cells.

### 3.5 *In vivo* pharmacodynamic studies

#### 3.5.1 Assessment of efficacy in MCF-7 tumor-bearing nude mice

**FIGURE 5:**
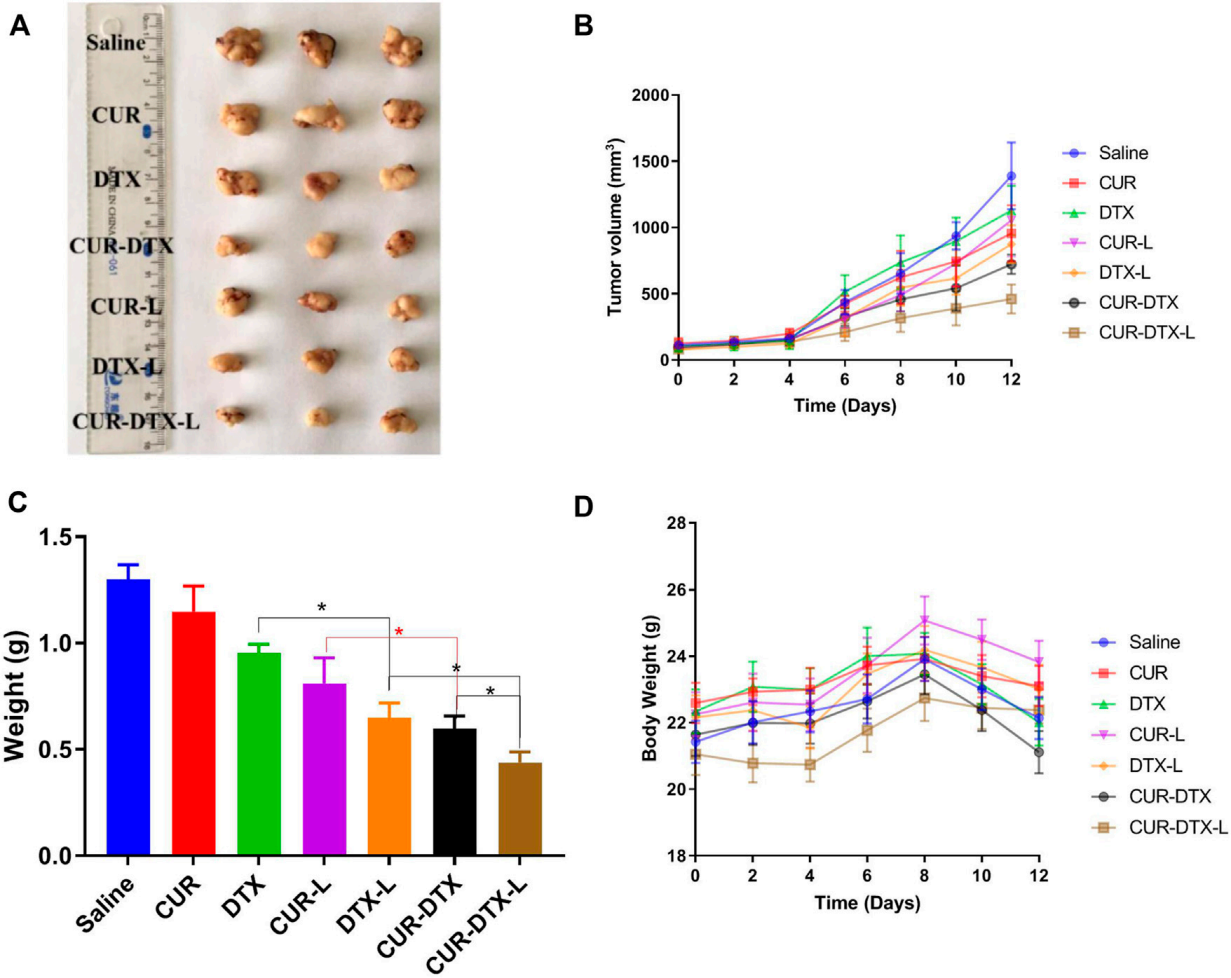
*In vivo* antitumor effect of tumor-bearing mice treated with saline, CUR, DTX, CUR-L, DTX-L, CUR-DTX, and CUR-DTX-L; *p < 0.05, **p < 0.01, and *n* = 3. **(A)** Tumor images of the different groups; **(B)** tumor volume curves; **(C)** weight of excised tumor tissues; **(D)** body weight changes of different groups during the whole therapy.

The anticancer efficacy of CUR-DTX-L was investigated using MCF-7 tumor-bearing nude mice, which can simulate human breast cancer’s proliferation and spread (Salem et al., 2020). As shown in Figure 5, the mean tumor volume in the saline-treated group increased more than 10-fold during the experiment. The results demonstrated that the tumor volume of mice treated with CUR was not significantly reduced compared to the saline group, whereas the tumor volume of mice treated with DTX was marginally reduced (Figures 5A,B). However, CUR-L, DTX-L, CUR-DTX, and CUR-DTX-L exhibit substantial antitumor actions and inhibit tumor growth significantly. Among them, the tumor volume and tumor weight of the CUR-DTX-L group were less than those treated with other medications (Figure 5C), and in Table 5, the IRT of the CUR-DTX-L group was the most significant at 66.23%, indicating that CUR-DTX-L inhibits tumor growth efficiently. Importantly, no substantial weight loss was detected in mice treated with CUR-DTX-L after the ninth day, in contrast to animals treated with other groups (Figure 5D), suggesting that CUR-DTX-L can mitigate the negative effects of CUR-L and DTX-L.

**TABLE 5:** Anti-tumor efficacy of tumor-bearing mice with saline, CUR, DTX, CUR-L, DTX-L, CUR-DTX, and CUR-DTX-L treatment (*n* = 3).

Group	Tumor weight (g)	Inhibition rate (%)
Saline	1.298 ± 0.07	—
CUR	1.148 ± 0.12	11.58
DTX	0.954 ± 0.04	26.50
CUR-L	0.811 ± 0.12	37.52
DTX-L	0.648 ± 0.02	50.80
CUR-DTX	0.597 ± 0.02	54.03
CUR-DTX-L	0.438 ± 0.14	66.23

#### 3.5.2 Histological examination and analysis

**FIGURE 6:**
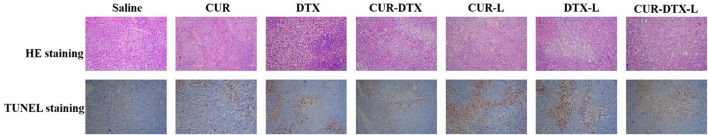
HE analysis and TUNEL immunohistochemical staining of tumor tissues (magnification ×200).

At the histology level, the anticancer effect of liposomes was studied using pathological sections. The top row in Figure 6 shows HE-stained tumor tissue from all seven groups. The treated group displayed a degree of necrosis. In the CUR-DTX-L group, tumor necrosis was the most severe. In contrast to the saline group, tumor necrosis was indicated by a lighter color in the other treatment groups. TUNEL staining revealed substantial differences as well. Rarely, tumors in the saline group exhibited immunoreactivity (brown). The single-drug-free group demonstrated incomplete positive spots (Shaik et al., 2004). Positive spots were observed in the CUR-DTX, CUR-L, and DTX-L groups. In contrast, the CUR-DTX-L group displayed several positive puncta in the form of dots. The cell death in tumor tissues can be efficiently reflected by TUNEL labeling. These results revealed that therapy with CUR-DTX-L could limit the growth of the tumor in a synergistic manner (Cai et al., 2021), which was consistent with the CCK-8 results.

### 3.6 Assessment of toxicity in nude mice

**FIGURE 7:**
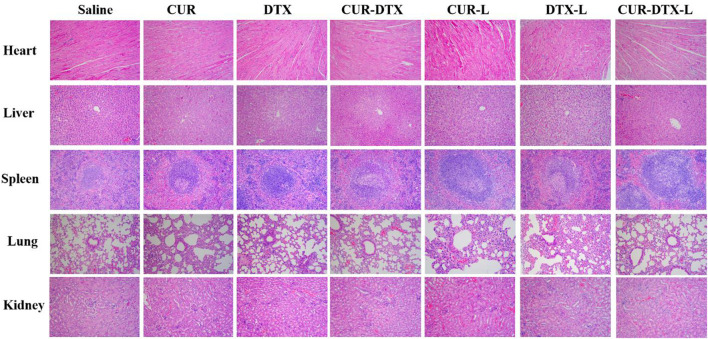
H and E staining images of the heart, liver, spleen, lung, liver, and kidney (magnification ×200).

The safety test was evaluated by HE staining of vital organs including the heart, liver, spleen, lung, and kidney, as shown in Figure 7. After treatment, there were only a few inflammatory cells infiltrated in the pulmonary alveoli, and interstation in DTX groups was consistent with previously reported results (Abbasi et al., 2015), with even attenuation in the combination treatment group. In addition, regardless of the control group, monotherapy group, or combination therapy group, there were no notable pathological alterations in other organs. HE staining demonstrated that CUR-DTX-L caused the most damage to tumor tissue, and no damage to the heart, liver, spleen, lung, or kidney was observed. In addition, Figure 5D shows that after 9 days, nude mice in the CUR-DTX-L group did not experience any substantial weight loss, in stark contrast to the weight loss reported in the other treatment groups. Consequently, CUR-DTX-L not only significantly enhanced the antitumor effect but also reduced their exposure in non-targeted tissues, therefore diminishing the overall toxicity *in vivo*.

## 4 Discussion

The combination of chemotherapy has been broadly studied in recent years, and combination therapy shows great potential in enhancing the antitumor efficiency, especially in MDR (Assaraf et al., 2019; Xu et al., 2019). Furthermore, because chemotherapeutic drugs are usually non-specific small molecules that have poor pharmacokinetics, they are only distributed in a non-targeted manner throughout the body, resulting in their reduced effectiveness at the actual target site. Therefore, the development of a delivery system that modifies the *in vivo* distribution of chemotherapeutic drugs enhances their deposition at the tumor site and reduces their side effects, which is a requirement for cancer-specific therapy (Wang et al., 2019).

In this work, DTX and CUR were co-encapsulated and delivered by the liposomes for the treatment of breast cancer. The physicochemical properties showed that the liposomes have the ideal size and zeta potential, which provided the benign passive accumulation of liposomes in the tumor site. Furthermore, for an ideal drug delivery system, the speed of drug release is critical. Over 95% of DTX was released from the free DTX group in 10 h and about 60% of DTX was released from the DTX-L and DTX-CUR-L groups after using a dialysis system after 48 h; although DTX cannot be dissolved in the water, about 60% of DTX in the liposomes was released after 48 h, indicating that DTX could diffuse through the dialysis membrane. This may be due to the crystallization of free DTX as the phase separates inside the liposomes and leads to the sustained release, and this kind of release could reduce the damage of organs responsible for the drug and lessen side effects (Pawar et al., 2018). In addition, the pharmacokinetics assays demonstrated that free DTX was rapidly metabolized and cleared *in vivo*, and the liposomes provided a foundation for long-term therapy of breast cancer and retention of drugs. The pharmacokinetic parameters of T_1/2_, AUC, and MRT also laid the groundwork for the accumulation and retention of drugs *in vivo* (He et al., 2019). These results are also consistent with those of other studies on DTX or co-delivery systems (Bowerman et al., 2017; Wang et al., 2019), which indicated that liposomes attenuated the rapid phagocytosis of the bloodstream by macrophages and prolonged the retention time of DTX and CUR in the body circulation.

The results of the CCK-8 assay showed that the liposomes could achieve better inhibition of cell growth in MCF-7 cells *in vitro* than the two free drugs alone and the liposomes encapsulated with CUR and DTX. Moreover, in order to assess the efficacy of CUR-DTX-L *in vivo*, we investigated the efficacy of the liposomes in MCF-7 breast cancer model nude mice. As shown in Figures 4, 5, the combination of CUR and DTX resulted in stronger anticancer efficacy than the two drugs alone. The results of the synergistic study of CUR with DTX were similar to those of previous reports (Hu et al., 2020; Tanaudommongkon et al., 2020), which may be attributed to the reversal of the MDR effect of DTX by CUR. At the same time, co-delivery allows for a reduced number of carriers with synergistic effects and relatively easy preparation compared to the respective encapsulated CUR and DTX. The synergistic anti-tumor effect of CUR in combination with DTX has been documented through the reversal of MDR by CUR [(Hu et al., 2020), (Sahu et al., 2016)]. But further studies are still needed to confirm whether CUR in CUR-DTX-L exerts synergistic anti-tumor effects by reversing MDR and, if so, what the mechanism for reversing MDR is.

Any drug has a dual nature, being both beneficial and detrimental to the body. To investigate whether CUR-DTX-L has an effect on normal organs, the differences in body weight and HE staining between tissues were used to evaluate the side effect. The results showed that the mice treated with DTX-CUR-L had no significant pathological changes, indicating that DTX-CUR-L not only has better anti-tumor effects but also has lower side effects. These results could further confirm the reduced toxicity and enhanced efficiency effects of liposomes.

## 5 Conclusion

In this study, nanosized liposomes called CUR-DTX-L were created for the simultaneous administration of DTX and CUR to breast cancer. The preparation method is straightforward and reproducible. Systematically, the particle size, PDI, and EE were evaluated.

Then, we evaluated the *in vitro* and *in vivo* behaviors of CUR-DTX-L. DTX and CUR were able to sustain release from liposomes compared to free drugs, and *in vivo* investigations revealed that liposomes may lengthen the drug’s retention period. The *in vivo* trial confirmed that the sustained release of medicine not only reduces side effects but also increases the drug’s anticancer activity. CCK-8 experiments demonstrated that CUR-DTX-L was able to reverse MDR and improve DTX-induced cytotoxicity and apoptosis. In the mice of the MCF-7 tumor model, CUR-DTX-L generated much larger tumor volume reductions and less systemic toxicity than other groups. On the other hand, CUR could reverse the drug resistance of DTX, and the liposomes may enhance the therapeutic efficacy of combination medications. Therefore, we believe that CUR-DTX-L can be a potential treatment for breast cancer.

## Data Availability

The original contributions presented in the study are included in the article/Supplementary Materials; further inquiries can be directed to the corresponding authors.
